# Military carrier choice during the Covid-19 pandemic in Hungary

**DOI:** 10.1007/s12144-022-02786-9

**Published:** 2022-02-11

**Authors:** Márta Pákozdi, György Bárdos

**Affiliations:** 1 ELTE Eötvös Loránd University, Doctoral School of Psychology, Lt. Colonel (OF-4) Hungarian Defense Forces, Budapest, Hungary; 2 Institute of Health Promotion and Sport Sciences, Faculty of Education and Psychology, ELTE Eötvös Loránd University, 1117 Budapest, Bogdánfy Ödön utca 10/B, Budapest, Hungary

**Keywords:** Covid-19 pandemic, Military service, Career choice, Motivations, Grounded Theory (GT)

## Abstract

Covid-19 pandemic has changed availability of reasonable jobs. To avoid joblessness, many young adults selected military service in Hungary. The aim of this research was to study their motivation, their individual needs and the way they think about the military forces. In this study, half-structured, focus group interviews with standard questions have been qualitatively analyzed by applying the Grounded Theory (GT) method. Three levels of coding were applied: *open* (basic), *axial* and *selective* coding. This process resulted in fewer codes representing larger categories leading to a final concentrated overview of the interviews. Three selective codes (Enrollment, Pathfinding, Fulfillment), and many sub-codes were identified. We could establish that the effect of the Covid-19 pandemic for the military career-choice really can be detected in many individuals. The military force offers for them safe, predictable existence and career image, in which, in addition to continuous learning and development, teamwork and camaraderie provides major motivations.

## Introduction

The aim of this study is to acquaint the reader with the motivation of those choosing the military service career in Hungary, and their relationship to the military forces and to their individual needs. The importance of this topic has increased recently since the interest toward the military career has been appreciated due to the worldwide Covid-19 pandemic. More and more people look for the Hungarian Army as for an employer (Ambrus, 2020), and more and more people with higher education (graduation, college, university) undertake crew position in the military forces. This publication shows the results of a study conducted in 2020 during the Covid-19 pandemic, focusing on the results of a qualitative research done solely within the crew. Hence, neither the Army itself nor the career choice of other national armies are included (Table 1).

**Table 1:** Need and/or motivation theories of career choice

AUTHOR(S)	MODEL NAME	ESSENCE	REFERNCES
Super	Life-career rainbow model	Personality and career requirements dynamically interact; main factors (immediate and perspective) are social structure, historical changes, socio-economic state, school, community, family. In addition, parents’ economy, mental state of the person and professional options also affect career	Super, 1955, 1957, 1990
Ginzberg	Decision series	Career choice is a series of decisions, in which the individual tries to seek consistency among his/her own goals and the reality of the world of work. This process is open ended and may last until finding a proper job	Ginzberg et al., 1951
Holland	Personality characteristics	Career choice is closely associated with personality thus people look for a job that fits the best to their personality characteristics	Holland, 1959
Armstrong and Murlis	Desire to be valuable	Everybody has a desire to be valuable. Internal motivating factors energize, control and maintain behavior, whereas external motives affect internal motivations, the goals which satisfies the individual needs and determine the series of states leading toward the goal	Armstrong and Murlish, 2005
Maslow	Theory of need hierarchy	It is necessary to satisfy the basic needs before moving on to the higher order needs. The peak of the need pyramid is self-realization, finding the internal potentials and using them. Human needs motivate to act, providing also an order for them. This, despite by many critics, is a very popular model	Maslow, 1943, 1987
Maslow critics	Maslow is incorrect?	Everybody have their own motivations, and the order of need satisfaction frequently turns to be not true	Hoffman, 1994; Kenrick et al., 2010; Diener & Tay, 2011; Sosteric & Ratkovic, 2020
Herzberg	Two-factor motivations	Two factors affect behavior as internal generators: *motivators* (performance, recognition, progress options etc.) on one hand, and *hygienic* factors (physical and structural characteristics of work environment and social work environment, respectively), on the other hand	Herzberg et al., 1959; Herzberg, 1966, 1968
McClelland	Learned needs model/ Need for Assessment Theory	Motivations in the social environment: *affiliation need* (relationship motivation, desire to be liked and accepted), *achievement need* (the success of internal drives to achieve and exceed our goals), and *power need* (affecting others – not a negative motivation, necessary for a certain extent to be a good leader). Motivations aren’t inherited but learnt, distinct in different societies, and may be developed by learning and training	McClelland (1961, 1987)
Atkinson—Oláh	Two factors affecting performance/Achievement motivation	Performance is affected by two factors: potential of success, and fear of lack of success. More success leads to more motivations, more failures result in less motivation However, attribution is an important additional factor of success. If the person feels being responsive for the failure, there is less chance to try again, whereas attributing to external factors may lead to more tries	Atkinson, 1974a, 1974b, 1976; Oláh, 2005
Hunt	Goal Contents Theory, GCT	Workplace behavior is directed by the goals, that may change through the life. This theory concentrates to the behavior and to the feeling satisfied with the repetitive behaviors, and not to the antecedents of the behavior and to the driving forces	Hunt, 1988
Csikszentmihályi	Flow and motivations	Flow is an extra state of the mental activity. Motivations are the result of experience of deepening and surrendering oneself to the actual activity, being involved into the process, pushing oneself for better performance, getting recharged and finding a joy in it	Csikszentmihályi, 1988, 1990, 2004

## Background—Theoretical

Since theories regarding military career choice are the same as for the general cases, we shortly summarize theories of career choice and their motivational background. Career choice is significantly affected by the immediate environment of the person (family, friends, school) as well as by the social influences (Allen, 2001; Beauregard, 2007; Super, 1955, 1957). It is an important factor whether the individual has adequate knowledge and notion about the selected profession. The non-adequate or low-level knowledge results in spontaneous, forced career choice (Allen and Russell, 1999; Auyeung and Sands, 1997).

Below we have collected some theories relevant for career choice.

## Background—Practical: Hungarian Army – Values, Norms, Socialization

To provide enough and correctly trained soldiers for the task performance of the Hungarian Army^1^ is a basic requirement. For this, direction toward the military career and career guidance is important, in which recruitment is one of the major factors.

The military service provides security, good existence and acceptance with continuous learning and development, teamwork and camaraderie, which provide significant motivations (Pákozdi, 2018). The difficult military service requires facing to challenges, like weapons management, driving and military practices, several training forms, and special operational trainings, and, in addition, mission participation and armed conflicts. However, the military career seems to be still attractive, and, due to the increasing joblessness^2^ initiated by the Covid-19 pandemic, the interest toward the military career has increased in both the Z- and even the Y-generations, respectively. In addition to the values and norms, livelihood and housing possibilities (in motels of the military organizations) are also attractive.

The first phase of the military socialization is the 6 weeks long basic training, in which the soldier acquires the basic theoretical and practical military knowledge and becomes capable to protect the homeland and the citizens. After completing the basic training, the life of the soldiers (individuals) changes, since they have to execute quite different tasks in new and different environment (both in social and localization terms) which represents significant load. The military socialization then is continuously evolving as a result of service in different military organizations or during armed and non-armed foreign assignments. It is an important feature that after Hungary has joined to the North-Atlantic Alliance (NATO) in 2004, her soldiers have to participate in the protection of other NATO member countries, thus Hungarian soldiers participate in NATO operations as well. As a member of the European Union, Hungary participates in EU missions, too.

Input requirements for entering the military forces are different in the different service categories. Minimal requirements for the crew members (soldiers) are primary school education or vocational exam. Due to the permanent development of the military technology, English or German basic language exam represents an advantage. In addition, unpunished life and physical and health fitness are required (all regulated by the Minister of Defense – HM regulation, 2020). It is important to note that higher education and higher-level language knowledge do not exclude accepting basic services if the individual accepts it and there are empty posts.

## Background – Antecedents: Interviews About The Military Career Motivations

The first author has initiated a series of military career-motivation interviews in 2019 among the incoming soldiers of the Hungarian Army. The interviews were taken during the first week of the basic training to learn more about their attitude and expectations toward the military forces, about the reasons for choosing the military services, and about their short- and long-term plans. The main aim had been to support them in adapting to the changing work conditions, new tasks, and different behavioral regulations. The interviews were followed by explaining and practicing the appropriate stress and conflict management techniques.

The interviews lasted for about 2 h in groups of 12 soldiers on the different sites and times of incursion. Based on the analysis of the military career-motivation interviews with 82 participants, it seems that the career choice of the crew members, in addition to the *security* and *regular income*, is associated with *patriotism* and *defense* of the *homeland*, respectively. From among the military values, *teamwork*, *respect*, *humility* and the intention to *follow* the *regulations* were also evident, although the latter evoked ambivalent feelings in some soldiers who were cautious to take the uniform being afraid of the *rigor* and *excessive regulations*. The *uniform* represented unity, dominance, power and strength for everybody. Their plans have generally focused on the military life, with an emphasis on learning and development and on mission assignments, whereas, in their private life, buying a home/house and starting a family were emphasized.

The motivation interviews have affected the development of the value-based organization culture of the Hungarian Army. Personality, thinking, behavior, opinion about the military forces, social capital and experiences about the basic training of the incoming soldiers not only enrich the organizational culture of the military forces but represent a society forming power through the values and opinions mediated by the soldiers.

Based on these former findings, the present study has aimed to explore and identify the military career-motivation of the incoming soldiers (crew) of the Hungarian Army by applying the *qualitative research design*.

## Methods

### Sample Collection Process

Participants had been selected from among the new soldiers of the Hungarian Army during the Covid-19 pandemic in 2020 on different places and different times. Enlistment has been open for the applicants even during the epidemic, although basic training has been done under strict pandemic rules.

Since military career-motivation conversations are regular elements of the basic training, selecting the sample was easy for a researcher who is also a military person. Selection has been arranged after a conciliation with the personnel office of the given military organization. Helped by the personnel offices, calls for the participation in the study had been delivered to the target group, describing the aim of the research, and the fact that participation is voluntary and anonym. It was also pointed out that only crew members (soldiers) can participate, despite the fact that sometimes non-commissioned and true officers may participate in the basic training.

### Data Collection

Data had been collected in groups of 12 soldiers enlisted during the Covid-19 pandemic in 2020 by a military career-motivation interview. Interview questions had been developed by the first author and were tested with 10 soldiers before this study. Replies to the semi-structured interview questions had been carefully noted. The questions were as follows:

- How have you become a soldier? Why have you chosen the military career?
  By using the words “how” and “why” we could achieve a deeper look at the motivation and attitude of the participants (Charmaz, 2008)
- What do you think about the advantage of the Hungarian Army as compared to other, civilian employers? What do you think about the disadvantage of the Hungarian Army as compared to other, civilian employers?
  From the answers about the advantages and weaknesses of the military forces we may get indirect information about the demands of the participants toward the military forces as well as about their knowledge of the military – that is with what they can identify themselves and what they expect from the military service.
- What does the uniform mean for you?
  Although uniform – in itself – is only a work garment, still can call many feelings that may inform us about their attitudes toward the military. We focused here also to learn about the expectations toward the military forces.
- What are your plans about your life?

Lifegoals have a deterministic role in one’s life, that require to set and realize short- and long-term plans. Getting to know lifegoals of the participants helps us to find out what other plans they think about in addition of those regarding the world of work.

### Sample

From the 229 persons enlisted for the interviews, 193 were males and 36 females, between 18–45 years of age (mean is 24 ys). 60% of them came from larger cities whereas 40% from villages. The participants came from all regions of the country, including West-, East- and Central Hungary, although due to the restrictive character of the applied Grounded Theory method, the sample is still not representative. Most of the participants completed vocational training, some graduated and two completed primary school. 4 officers and 6 non-commissioned officers had been excluded since being a soldier (crew member) had been a basic criterion of the participation.

Talks had been arranged in groups of 12 soldiers wearing uniforms, lasting for about 2 h in a semi-circular arrangement (Fassinger, 2005; Ponterotto, 2005, 2010). During the talks, the moderator, as a conductor, has omitted the military rules (O’Donell, 1988), like command, I report, etc. which significantly contributed to formulate true and honest replies (Corbin & Strauss, 2008, 2015; Krueger, 1988, 1993). The focus group arrangement also made possible to identify the attitude toward and thinking scheme about the military forces (Barbour & Kitzinger, 1999). Altogether, notes of 229 interviews (19 group notes) have been analyzed by applying the Grounded Theory (GT) method (Charmaz, 2008; Corbin & Strauss, 2015). Participants had been clear in formulating their opinion, feelings and thoughts regarding the obtained questions. Social characteristics of the respondents had not been examined; however, gender and age were recorded.

### Data Analysis

To learn about the feelings and thoughts of the soldiers about career choice, military forces, uniforms, and, in addition, to know more about their lifegoals (short- and long-term plans), we used the GT analysis (Charmaz, 2008; Corbin & Strauss, 2015). The qualitative analysis of these data offers finding and formulating new views and correlations, to rearrange earlier knowledge, and to reformulate meaning of existing terms (Corbin & Strauss, 2015). By systematic analysis of the data, it may also be possible getting to produce a relevant theory (Corbin & Strauss, 2015).

During the analysis, three levels of coding were applied: open (basic) coding, *axial* coding and *selective* coding. This process resulted in less and less codes representing larger and larger categories leading to a final concentrated overview of the interviews (Corbin & Strauss, 2015). During the analytic process following several read overs, we first created open codes by identifying significant elements. During the axial coding process, we intended to explore the connections among the open codes and the different dimensions. In this period, we have made several notes and memos which has helped our work significantly. Finally, during the selective coding we compared the set code system to the original text, have refined the code system, fitted to the text, and finally have formulated the final (selective) codes (Corbin & Strauss, 2015). The code-families were depicted as diagrams to help the reader understanding the complicated connection system of the codes. Coding has been done by the first author, then it was discussed. Since there wasn’t any disagreement, further action has not been warranted.

## Results

### Codes

**Fig. 1:**
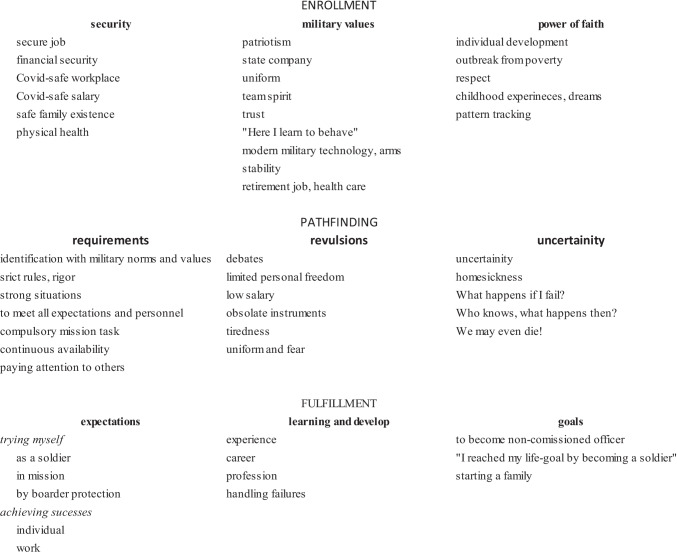
Career-choice selective (in capitals), axial (in bolds) and open codes

Figure 1 shows the three selective codes and the associated relevant terms (axial and open codes) obtained in the GT analysis:

The analyzed texts represented military career-choice factors, which are commonly characterized by the fact that the soldiers had described their motivations as a consequence of external force. Most of the texts are in first person singular, but the social experiences are in plural. The interviews frequently looked backward to the past, remembering childhood experiences, and to the future, as well, with outlining their plans.

The analysis has revealed three selective codes extracted from different sub-categories.

The selective code ENROLLMENT is present in the reports as a basis, consisting of three sub-categories (i.e. axial codes), on the first-place security, followed by military values and then by power of faith.

Security, as one of the basic motivations for enrollment, mentioned by all respondents, contains *secure job*, *financial security* and *safe family existence*. In addition, we have found sophisticated description of categories associated with the Pandemic, like *Covid-safe workplace* and *Covid-safe salary*, and *physical health*. In addition to meaning a basic motivation, security at the same time provides also stability of their life.

The second axial code, the military values, includes *patriotism*, characterized by expressions like protection of the homeland, appreciation, endurance, pride, bravery, determination, and obedience. *Team spirit* (also a military value) has been uniformly mentioned by all participants, together with *trust* associated with camaraderie. There is a major difference among those who entered earlier (2019) and those enlisted during the pandemic: whereas the formers regard protection of the homeland as a primary motivation, for the latter it is only a secondary motivation. *Uniform*, which in itself represent a significant and universal value, is associated with *stability* and *state company*. The sentence “*I learn here how to behave*!”, associated with the compliance with the norms and rules was also mentioned by several participants. The *modern military technology and arms*, as motivating factors, are also present in several reports. Interestingly, the *retirement job* and *health care* were mentioned relatively rarely, probably reflecting to the fact that the new soldiers do not look too far ahead at the moment.

The military career as a chance to *outbreak from poverty* appears as an important element of the axial code power of faith, an example value for everybody. The desire for *individual development* was also present in the reports referring to individual plans and goals, together with *respect*, given and obtained as a result. Several participants mentioned the role of *childhood experiences*, for example those obtained in military camps or military competitions. Childhood dreams were mentioned here, too, which some mentioned as a goal to realize in adulthood. Although with low frequency, but very strongly were mentioned the *pattern tracking* of parents’ or friends’ (social) behavior, effectively associated with military profession.

**PATHFINDING** is the selective code of obstacles and dams, associated with all sub-categories which may result in or promote disarmament during the basic training or later, too. Several ambivalences associated with sub-categories mentioned under the ENROLLMENT code can be identified under this selective code, too. Such sub-codes are *military values* versus *strict rules* and *rigor*; *modern military technology* versus *obsolete instruments*; *uniform* and *fear*; *uniform* and “*We may even die*”; the *power of faith* versus “*Who knows what may happen*?”; *financial security* versus *low salary*; *stability* versus *uncertainty*. Several participants have mentioned the *limited personal freedom*, the *continuous availability*, and the *homesickness*. *Tiredness*, *debates*, *paying attention to others* are also interesting terms, even though mentioned rarely. The fear associated with uniform, as well as the thought “*We may even die*” is characteristic for those having been enlisted in the second half of 2020, together with the uncertainty, tiredness, hopelessness, rigor and homesickness.

**PATHFINDING** means that the new soldiers face a lot of requirements, like *strict rules* and *rigor*, *strong situations*, and *compulsory mission tasks*, typical characteristics of the military service. They also realize that they must *identify* themselves with *the military norms and values*, to *meet* all *military* and *personnel expectations*, to be *continuously available* for service and to *pay attention to others*.

The strong requirements they realize mostly just after becoming soldiers frequently generate **revulsions**. On the wrong side of the picture they may find *limited personal freedom*, *low salary*, *obsolete instruments*, factors just against their expectations, together with *debates* and *tiredness*, that is behavioral difficulties. These may overshadow the positive meaning of the *uniform* by associating it with *fear*.

Those who had hoped to be safe entering into the Army now may find **uncertainty**. Expressions like ‘*What happens if I fail?*’, ‘*Who knows what happens then*?’ mirror essential fear of failure, while exclamation ‘*We may even die*!’ shows the fear of the worst possible outcome.

**FULFILLMENT** is a selective code displaying plans and goals. The elements of this code can be reached based on ENROLLMENT and by passing the obstacles of PATHFINDING. Some participants formulate it in past tense “*I have reached my life-goal, have become a soldier*”, whereas others look to the present and also forward to the future (“*I have been preparing for this, I want this*”). For the minority of the participants, finding *experience* is imagined through completing missions, whereas the majority rather tries it through completing the everyday tasks.

**Expectations** may set the path to follow, one to *try myself* and another *achieving success*. Trying oneself includes *as a soldier*, even *in mission* or *by boarder protection*, whereas success *individually* in the *work*. The way to reach the goals require learning and develop throughout the *career*, acquiring *experiences*, building up a *profession* and by *handling the failures*. These all may lead to the FULLFILLMENT of goals. There are ones already fulfilled ‘*I reached my life-goal by becoming a soldier*’, others are future plans, like *to become a non-commissioned officer*. If expectations become true, one of the final goals and a basic motivation, *starting a family* could be met.

### Overview Of The Codes

The dominant basic code of military values, the *uniform*, represents uniformity which is able to eliminate social differences thus providing a chance for *development* and growth. This seems to be evident for the soldiers since they express their need for *learning* (Herzberg, 1968; Maslow, 1943). The power motivation associated with the uniform also appears in the form of symbolizing the protection of the homeland, an obligation of the soldiers – “*I protect my homeland, I am preparing for that*”. The *trust* is represented by the military as an employer, which one can rely on under any circumstances, and by the *teamwork*, which represents collective knowledge as well as emotional security (McClelland, 1987; Peterson & Seligman, 2004).

While thinking of the motivations associated with the uniform, some soldiers have shown the positive change of the signs of depression and despair due to the joblessness, one could observe serenity, gratitude and curiosity on their faces.

The *need for compliance* and the *new military technology and arms* show an ambivalent picture. For the majority, these are positive motivating factors, although some mentioned them as weaknesses of the military; the background of which probably being the lack of knowledge and stereotypes (Auyeung and Sands, 1997). The definitive rules and commands, which organize the daily life, communication and task execution give a sort of frame for the soldiers, make their life easier, help to orient themselves in the everyday life (Cesar, 1995; Eidson, 1995). Most of the soldiers require learning and appreciating behavioral patterns; “*Here I learn how to behave*”, frequently filling a gap, too.

Outbreaking of poverty is a so strong motif for some soldiers, that can be interpretable only via the *power of faith*. They brought it from their childhood, also present in their young adulthood. For them, the uniform means clean clothes, the three daily meals mean staying alive, the crew hostel means shelter. It is, however, important to mention that they also appreciate learning, appreciation, need for development and patriotism as well as dedication to protect the homeland.

The dominance of the need for being *appreciated* (Maslow, 1943) is associated with the uniform and with the values represented by it. After having unpleasant feelings in other workplaces, soldiers feel and believe that the Hungarian Army, as an employer, respects them and provides a track for continuous development (Maslow, 1943) and also recognizes their drives to protect the homeland (Herzberg, 1968): “*Here we are respected and could develop*”; “*What we do, is appreciated*”. This sub-category looks standing out of the others, and in itself seems to be able to win over the sub-categories identified under PATHFINDING, and may lead to self-realization (Maslow, 1943). For the personal development, effective service performance and professional activity, appreciation and recognition of the soldier look essential. Without them, the soldier questions his/her existence in the system, self-esteem becomes reduced, he/she falls back to the level of basic needs which may lead to changing the assignment and finally to *discharge*.

Under the selective code PATHFINDING new codes not found in earlier research (2019) appear, like tiredness, paying attention on others, discussions, probably accompanying the permanent joblessness (Jahoda and Rush, 1980, Kawohl & Nordt, 2020) in the case of some newcomers as a consequence of low self-esteem. Homesickness shows up during the first week of military socialization through the feeling of leaving ordinary persons and objects, and if intensifies, may lead to discharge, representing also the loss of life goals.

The feeling of *restriction of the personal freedom* refers to the being informed and also to the inability accepting it, which is seemingly another reason for thinking of disarmament. The mentioned *low salary* is not equivalent with the *Covid-safe salary*, which is although the basis of livelihood, still reflects to the fact that the soldier is expecting more income from the army for the time being, making starting and supplying a family possible. In the case it should not happening, he/she may think of disarmament.

Goals and plans present in the FULFILLMENT code (Hunt, 1988) serve as drives for the soldiers toward the commitment of the military service, the source of what, in addition to financial security, is their need for recognition and self-development (Maslow, 1943) and their belief in the military values. The real goal seems to be becoming a non-commissioned officer, the building of a career and formatting a profession. Becoming a non-commissioned officer is associated with the military profession, thought to be a somewhat slower process than the building of a career. According to their views, the focus is on the longer learning, experiencing and developing procedures, during which the military track may become their profession. According to them, career is a shorter goal, which means about 10 years of acquiring knowledge and proficiency, including mission services, too, and eventually leads to leaving the track. They imagine using their knowledge on other tracks, like personal bodyguards. This represents advantages and also disadvantages for the Army.

Searching for experiences (Csíkszentmihályi, 2004) and testing themselves, stretching their limits in unfamiliar situations, places and tasks color their life and push them toward reaching more achievements (Atkinson et al., 1976). Curiosity behind the experience search includes openness to new things, critical thinking and love for learning. This so-called *experience searching character* also contains the need for (financial and emotional) security, for which, however, they would be able in non-military conditions, too. These soldiers want to experience. develop, grow, and are not really motivated by material advancements (“*I am not that much interested in the money*”) but to comply with the challenge. They are not necessarily rule followers, but still complete the tasks the best way they could, using bravery and creativity. It is difficult to keep them if their self-development is inhibited and they go on to look for better challenges (employers) (“*Earlier I had served for the police but could not have developed enough*”).

## Discussion

The participants have presented their motivations for the career-choice in a sophisticated way, sometimes even by non-verbal signs (anger, sadness, hopelessness, gratitude, enthusiasm). It is true that security and the regular income for normal lifestyle already had appeared as basic needs (Maslow, 1943) during the military career-choice of the participants, however, in addition, patriotism, protection of the homeland and identification with the military values (teamwork, respect, humility and compliance) as elements of the conscious career-choice (Pákozdi, 2018) as motivations had also been present (Borgen & Hiebert, 2006, Ginzberg et al. 1951, Holland, 1959, Super, 1955, 1957, 1990).

After analyzing the interviews with the soldiers, we can establish that the effect of the Covid-19 Pandemic for the military career-choice really can be detected in many individuals. For the newcomers, during the pandemic– mostly youngsters between 20–30 years, some between 30–35 years -, however, the desire for satisfying the security need appears emphatically, which they express by using the word “Covid”, like “*Covid-safe salary*” and “*Covid-safe workplace*”. The Covid expression suggests military career-choice directed by external factors (Borgen & Hiebert, 2006, Ginzberg et al. 1951, Super, 1990).

Decision of those not having enough knowledge about the military track (Borgen & Hiebert, 2006) are primarily motivated by the *subsistence of themselves and of their family*, again representing an external goal-activation for the individuals (Jahoda and Rush 1980) reinforced by moving toward a state company. Their desire for a secure job is supported by their earlier experiences with other, civilian workplaces (Hunt, 1988): “*We hadn’t got our salary in time*”; “*They haven’t given money*”; “*We weren’t announced*”. They imagine all these can be correctly done by the military, “*Army is always necessary*”, “*Army always had been and will also be forever*”, which may result in their identification with or in the refusal of the military values.

These are reflected by their words: *rigorous rules, discussions, to meet all requirements and all persons, tiredness, uncertainty, fear* associated with *the uniform*. The loud exclamations, like “*We even may die*!”, or the work without correct plans “*Who knows what happens then*?” appear as obstacles within the PATHFINDING and may work against the commitment toward the military track and the military profession.

It is, on the other hand, true, that *uniform*, a primary military value, sub-category (axial code) of ENROLLMENT in itself provides security and stability for them: “*It is a good feeling to wear it*”, “*I feel it protects me against everything*”, “*It provides protection and security*”. For them, wearing the uniform also means belonging to somewhere (Maslow, 1943; McClelland, 1961, 1987) – “*It is a good feeling to be the member of a team*”, “*It is a good feeling to belong to somewhere*” -, thus the military service gives a frame and stability for their life. Self-esteem of most of the newcomers becomes associated with winning the obstacles identified in the PATHFINDING and with the realization of their goals. It functions as a drive and relies on an internal conviction.

Family pattern tracking (parents, siblings) and social impacts (friends, acquaintances) (Super, 1955, 1957, 1990, Jahoda and Rush 1980, Csikszentmihalyi 1990), as basic codes of the ENROLLMENT are frequently present in the reports, supposedly referring to high level knowledge of the system – “*We have got military education from our childhood*”; “*In my family, everybody is a soldier*”; “*My friends have told a lot about the military life*”. Childhood desires are also present in the sample, referring to commitment toward the military – “*I have wanted to become a soldier from my childhood*”; “*I have reached my goal becoming a soldier!*”.

Experience search contains such events like military practices with good equipment (clothing, new arms), day and night, in the mud, in sunshine, in rain, in wind, associated with such events like “*Jumping into and from a tank*”, and with handling *new military equipment*. For those looking for new experiences, trying the military existence is the first step, onto which mission engagement (armed foreign services) and protection of the boarders against immigrants can be built. These tasks, in addition to emotional and physical load, also provide higher salary representing a strong motivation, too. They feel this way they could be able to get the necessary financial resources, the existence for starting a family.

The individual and work success support them as inner forces (McClelland, 1961, 1987) in achieving their goals and in their service commitment. Becoming a non-commissioned officer is an open possibility, an available goal for many of them (Hunt, 1988). To achieve it, they need to extend their practical and theoretical knowledge, as well as self-confidence, endurance and good management of their failures. The sentence in past tense “*I have reached my goal by becoming a soldier*” formulates a satisfied goal encouraging the individual for better performance and for reaching more success.

The general goals appearing as sub-codes of the selective code FULFILLMENT reflect to the large-scale plans of the soldiers, although not always based on real knowledge about the military forces. If their socialization, life-situation become stable and improve, their military goals may expand and change, if they possess enough energy.

### Symbolic Analysis Of The Selective Codes

Each selective code represents symbolic meaning by which it seems possible to describe motivations for military career-choice. We feel it is important to show them, and, in addition, our aim is to contribute to the development of GT analysis.

**Fig. 2:**
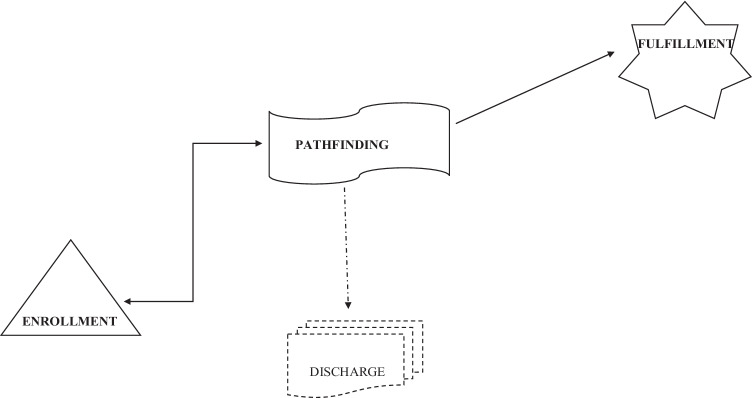
Selective codes network

From the symbolic meaning of the selective codes (Fig. 2) the ENROLLMENT located within an isosceles triangle is present in each report as a basis. The pedestal of the triangle provides stability for the individuals when choosing a career and, in addition, supports the decision from each side. The values grouped here based on individual decisions, like *uniform, patriotism, secure job, team spirit, security*, serve as ENROLLMENT and help the person in military socialization. The peak of the triangle projects ahead the individual goals, *like mission assignment, trying-oneself, potential to learn and develop, achieving success, start a family, getting existence, career and profession*, which come together in the selective code FULFILLMENT. The peak of the triangle also represents the time required to achieve the goals, and the facts, too, that to reach them is accompanied with many decision situations, choosing and ordeals. These decision situations, obstacles come together in the PATHFINDING waving symbol. The route to the win–win situation leads through the power of faith, that is through the belief-in-oneself (“*I am able to do it, I can do it*”; “*I am able, I can reach my goals*”). The “visionary code” *discharge* originates from the PATHFINDING selective code, is represented by an icon showing a series of visionary doors. This is the route of the *dismantlers*, the door opening as the consequence of unsuccess of bypass the obstacles, when the ENROLLMENT is not enough while PATHFINDING. It is a fact, however, that the time spent in the army affects the further life of the individual, symbolized by the series of doors.

Arrows connecting the symbols represent the relationships. The two-way arrow connecting the ENROLLMENT and the PATHFINDING represents the continuous fight with the obstacles and dams, in which the ENROLLMENT will be the winner.

The arrow from the PATHFINDING symbolizes achieving the FULFILLMENT, in which the nourishing role of the ENROLLMENT is indisputably present. The dashed line arrow originating from the PATHFINDING selective code leads to the disarmament, to the projected way of those deciding to leave the track. For them, the power and charge of the ENROLLMENT are not enough. It is still not clear, however, which ENROLLMENT codes should need for the way back, that, not being a topic of this study, may require further research.

### Strength and Limitations

The major strength of this study is the application of qualitative analysis (i.e., the Grounded Theory method) which uncovered many facts and ideas unknown before. An advantage of this method is that the participants may express their full opinion freely, not restricted by the questions as in the quantitative studies. And really, even hidden fears (like ‘We may even to die’) appeared clear during the interviews.

The major limitation is the relatively low number of participants, which is a disadvantage of the GT method. Although this way the sample is not representative, still very useful since the participants had come from all parts of the country.

## Conclusions, Recommendations And Future Direction

This study has shown that the Covid-19 pandemic represents a threat especially for the young adults regarding the availability of jobs. Many people have lost their job and had to look for new opportunities. In this situation, the military service in the Hungarian Army represents a readily available and promising option, offering secure, lasting and good conditions, reasonable salary and wide range acceptance. The new soldiers learn adapting to the requirements, recognize the perspectives and set reasonable goals. Although some of the newcomers have difficulties, which may lead to disarmament and discharge, the majority accepts the challenges and stays in the service.

It seems that based on these findings, the Army may invite more new applicants, offering security, good circumstances and promising life goals. Development of new technology, up-to-date armament and widened service options may help to the new soldiers to accept the invitation and to become satisfied military servicemen.

These results also help to appoint new directions for the research. One promising way is to follow the life and service conditions of the soldiers participating in this study to see how the promises will be satisfied and how they will feel after a while. Another interesting question is the possible diversity of the new soldiers regarding age, gender and education, their capacity to accommodate and overcome the difficulties.

## Data Availability

Data are available on request from the authors. The data that support the findings of this study are available from the corresponding author, upon reasonable request. The authors declare that there are no conflicts of interest which could pose a real, possible or apparent problem regarding their participation in the publication. Furthermore, the first author declares that as a soldier she is a member of the Hungarian Defence Forces, however, this does not have any impact on the findings and conclusions of the study. Moreover, she also declares that the findings and conclusions of the research are exclusively based on what was said by the respondents. The second author declares that he is not a member of the Hungarian Defence Forces and has no connections with the Hungarian Defence Forces that might have any effect on the findings and conclusions of the research.

## Footnotes

1 Based on an open source, the number of employees in the Hungarian Army is 26 753 prsns (https://index.hu/belfold/2019/03/19/magyar_honvedseg_zrinyi_2026_harckocsi_leopard_2_onjaro_loveg_airbus_helikopter/

2 According to the National Statistical Office, population of Hungary at September 2020 was 9 769 526 whereas the joblessness was nearly 222 000. (https://www.ksh.hu/docs/hun/xstadat/xstadat_eves/i_wdsd003c.html?down=1219https://www.ksh.hu/docs/hun/xftp/gyor/mun/mun2009.html;
